# Geography and Host Identity Shape Intraseasonal Variation of Free‐Living and Zooplankton Associated Microbial Communities in Alpine Lakes

**DOI:** 10.1111/mec.70069

**Published:** 2025-08-12

**Authors:** Christopher B. Wall, Madeline G. Perreault, Margaret Y. Demmel, Evelyn M. Diaz, Joshua H. Dominguez, Jonathan B. Shurin

**Affiliations:** ^1^ Department of Ecology, Behavior and Evolution, Division of Biological Sciences University of California San Diego California USA; ^2^ Department of Earth Sciences University of Hawai‘i at Mānoa Honolulu Hawaii USA

**Keywords:** bacterioplankton, copepod, *Daphnia*, microbiome, Sierra Nevada

## Abstract

Microbes contribute to aquatic ecosystem function and the fitness of macroscopic organisms, including zooplankton. Many factors affect the taxonomic compositions of free‐living (bacterioplankton) and zooplankton‐associated microbial communities in lakes; yet how these communities vary seasonally and among lakes remains poorly understood. Here, we investigate how free‐living bacterial communities and those associated with different crustacean zooplankton hosts change in response to fluctuations in their natural environment across time and space. We repeatedly sampled bacterioplankton, zooplankton communities, zooplankton microbiomes, and water chemistry parameters of six lakes in the eastern Sierra Nevada mountains of California across a summer season. 16S rRNA gene sequencing revealed clear differences in the community composition and relative abundance of bacterial taxa between bacterioplankton and zooplankton microbiomes, which was best explained by lake and host identity rather than intraseasonal sampling times. Bacterioplankton communities were highly conserved across the summer season and showed higher alpha diversity, but lower species turnover, than zooplankton microbiomes, which were more variable and largely partitioned by host taxa and phylogenetics (Copepoda vs. Cladocera). Spatial and local environmental context (drainage basin, home‐lake habitat) interacted secondarily with community types (free‐living, host‐associated) and zooplankton host identity to shape bacterial community composition. These results show that deterministic processes related to host filtering, host taxonomy, and spatial/environmental variation among lakes drive changes in microbial communities more than temporal changes within lakes. Higher beta diversity among zooplankton‐associated microbes suggests dispersal limitation and/or local selection play stronger roles for zooplankton microbiomes than for free‐living bacterioplankton.

## 1 Introduction

Microbes are ubiquitous in freshwater environments and play key roles in ecosystem function and the fitness of host organisms (Cavicchioli et al. 2019; Gupta et al. 2017). Bacteria have long been considered as recyclers and remineralisers of organic material in lake ecosystems (Azam and Ammerman 1984; Lindeman 1942; Newton et al. 2011), and prokaryotes affect processes throughout lake food webs at every trophic level (Cavicchioli et al. 2019; Heino et al. 2021; Newton et al. 2011). In addition to living freely in the environment, microbial communities live on and within multicellular host organisms (Adair and Douglas 2017; Akbar et al. 2022), collectively the ‘holobiont’, where the function of multicellular hosts is intimately connected to their associated microbes (Grossart et al. 2013; Vandenkoornhuyse et al. 2015).

In aquatic ecosystems, zooplankton and the diverse consortia of microbes that associate with them (hereafter, ‘microbiomes’) are essential members of food webs that influence lower trophic levels through grazing (Eckert and Pernthaler 2014; Wang et al. 2021). Together, free‐living bacterioplankton and zooplankton are vital to nutrient cycling in freshwater systems and serve to transfer energy to higher trophic levels (Barnett et al. 2007; Cotner and Biddanda 2002). The assembly of microbial communities (both free‐living and host‐associated) follows deterministic (niche‐based) and stochastic (neutral‐based) processes and is a product of abiotic selection, species interactions and chance events (e.g., random drift, colonisation, extinction) (Aguilar and Sommaruga 2020; Chase 2010; Macke et al. 2020; Stegen et al. 2013). The relative importance of these processes in shaping microbial communities can differ across short‐ and long‐term scales and across free‐living and host‐associated habitats (Hegg et al. 2021; Stegen et al. 2012; Vass et al. 2020). For instance, stochastic processes may best explain bacterial community composition at local and regional scales (Roguet et al. 2015) and over short time periods (Aguilar and Sommaruga 2020). However, deterministic processes and environmental filtering can play important roles in shaping bacterial community composition in response to seasonal changes (in abiotic conditions and metacommunity succession) (Kent et al. 2004) and across lakes over long‐term scales (Aguilar and Sommaruga 2020).

Freshwater zooplankton communities are influenced by biotic (fish predation and phytoplankton availability) and abiotic factors (light availability, temperature and water chemistry) that change seasonally (Özçalkap and Temel 2011; Pinel‐Alloul et al. 1995). Seasonal changes in environmental conditions in aquatic ecosystems are expected to shape the composition of both free‐living bacterioplankton and zooplankton‐associated microbiomes. For instance, free‐living bacterioplankton communities in lakes of the Sierra Nevada mountains are influenced by temperature, dissolved organic carbon (DOC) chemistry and latitude (Schulhof et al. 2020). In lakes of the US Rocky Mountains, early summer differences in bacterioplankton communities between subalpine and alpine lakes correlated with DOC, chlorophyll and dissolved nitrogen driven by hydrologic connections to watersheds (Vincent et al. 2021). In eutrophic lakes, bacterioplankton communities vary seasonally and follow a predictable phenology across years with environmental correlates (dissolved oxygen, nitrate+nitrite and temperature) related to cycles of mixing and stratification (Shade et al. 2007). Similarly, zooplankton microbiomes may also respond to environmental conditions, including food availability, water temperature and the presence of toxic cyanobacteria (Akbar et al. 2021; Eckert et al. 2021; Frankel‐Bricker et al. 2020; Sullam et al. 2018). During repeated summer sampling of *Daphnia* from mesocosms in an eutrophic lake, the relative abundance of taxa in the *Daphnia* gut microbiome significantly changed over time, indicating high plasticity in zooplankton–microbe associations (Hegg et al. 2021). Therefore, while bacterioplankton communities follow seasonal and long‐term changes in climate, temporal variation may independently affect zooplankton microbiomes (Hegg et al. 2021; Shade et al. 2007). Yet, the magnitude of temporal variation and flexibility of the zooplankton‐associated microbiomes in relation to spatial turnover among lakes, or among different zooplankton hosts within lakes remains unknown.

Zooplankton microbiomes are influenced by horizontal transmission of free‐living microbes from the ambient water (Akbar et al. 2022; Macke et al. 2020; Mushegian et al. 2019). In *Daphnia*, the lake environment provides an important source of microbial colonists. For instance, gut microbiome diversity in laboratory‐reared zooplankton increased after being translocated to in situ enclosures (Hegg et al. 2021). Similar microbiomes have also been found across taxonomically distinct zooplankton hosts (cladocerans, copepods and rotifers), with dominant bacterial taxa matching those found in high abundance in the surrounding water (Eckert et al. 2020, 2021; Hegg et al. 2021). However, consistent dissimilarity between zooplankton microbiomes and free‐living microbial communities indicates that host selection/filtering plays a key role in structuring zooplankton microbiomes (Hegg et al. 2021). Despite much progress in recent years, the factors that shape the assembly of free‐living and zooplankton‐associated microbial communities in natural settings remain poorly resolved.

Alpine lakes of the Sierra Nevada are known to support highly functional ecosystems and provide many crucial ecosystem services such as freshwater supply for endangered habitats and human populations (Null et al. 2010). However, these lakes are increasingly vulnerable to the impacts of climate change, including higher temperatures, longer and more intense droughts and reduced snowpack, all of which could lead to the disruption of food webs and nutrient cycling, and the decline of water availability and quality (Lopera‐Congote et al. 2024). Here we ask how free‐living bacterioplankton and zooplankton‐associated bacterial communities of common crustacean hosts vary intra‐seasonally within six alpine lakes in California's Eastern Sierra Nevada mountains. Alongside sampling microbiomes at five time points, we measured physio‐chemical properties associated with water quality and lake productivity (lake surface water temperature, pH, dissolved oxygen, conductivity, dissolved organic carbon, total dissolved nitrogen and phosphorous and chlorophyll *a*) (Bhateria and Jain 2016). Zooplankton are sensitive to fluctuations in the environment, and their biomass and composition can be an ecological indicator of water pollution and other aspects of lake health (Li et al. 2017; Xu et al. 1999); therefore, we also monitored the densities and community composition of zooplankton communities in lakes across time. We use 16S rRNA gene sequencing of bacterioplankton and zooplankton microbiomes to characterise variation in composition associated with host species identity, lake habitat and time points. We hypothesise free‐living and host‐associated bacterial communities would be dynamic through time, driven by changes in environmental conditions (Hegg et al. 2021; Schulhof et al. 2020), with lakes in close proximity showing greater bacterial community similarity. Freshwater zooplankton have been shown to display low specificity in microbial associations (Eckert et al. 2021); therefore, we predict similar zooplankton microbiomes across taxonomically diverse hosts. Our goal was to partition bacterial community diversity and composition into spatial (lake), temporal (date) and habitat (host‐associated vs. free‐living) components to understand the drivers of composition in lake bacterial communities across scales of space and time.

## 2 Materials and Methods

### 2.1 Study Region and Lake Information

**FIGURE 1:**
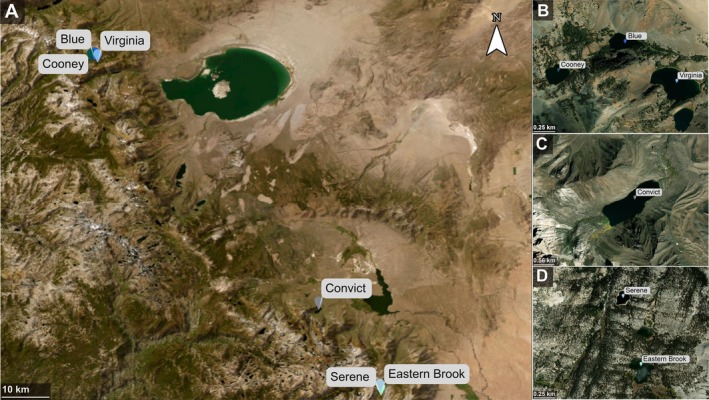
(A) Geographic locations of the six mountain lakes, located in the eastern Sierra Nevada mountains, CA, USA. Pins indicate lake locations and elevation, with subpanels (B–D) detailing the three sampling zones. Map source: A: Earthstar Geographics, B–D: Maxar, Microsoft.

We sampled zooplankton, water and biogeochemical variables in six lakes in the Eastern Sierra Nevada mountains of California, USA: Eastern Brook, Serene, Convict, Cooney, Blue, and Virginia (Figure 1). Lakes were sampled every 2 weeks from 25 June to 23 August 2022, for a total of five sampling time points (Table 1). To complement the environmental and water chemistry metrics measured in each of the six lakes across the five sampling time points, we recorded average air temperature and total daily precipitation from Sierra Nevada Aquatic Research Laboratory (SNARL) in Mammoth Lakes, CA, USA (Figure S1).

**TABLE 1:** Site information for six mountain lakes sampled in the Eastern Sierra Nevada mountains.

Lake	Latitude	Longitude	Elevation (m)	Depth (m)	Zone	Fish
Cooney	38.04806	−119.27702	3116	14.0 ± 0.5	Alpine	Yes
Blue	38.05095	−119.27033	3005	14.7 ± 0.4	Alpine	Yes
Virginia	38.04698	−119.26508	2983	15.7 ± 0.6	Alpine	Yes
Convict	37.59009	−118.85742	2315	42.9 ± 0.6	Montane	Yes
Serene	37.43839	−118.74439	3124	7.1 ± 0.1	Alpine	Yes
Eastern Brook	37.43153	−118.74262	3155	8.0 ± 0.6	Alpine	No

*Note:* Depth is mean ± SE across six sampling periods measured at the deepest part of the lake. Fish indicates the presence or absence of fish in lakes.

### 2.2 Lake Sampling

We sampled lakes from a kayak anchored at the deepest part of the lake. We deployed a calibrated YSI Pro Plus Multiparameter Instrument (YSI Inc.) at 1 m below the surface to record surface temperature, pH, conductivity and dissolved oxygen percent (DO). We sampled lake epilimnion (surface to 1‐m depth) using a tube sampler and filtered the water through a 64‐μm mesh into two carboys. We filtered water through pre‐combusted (550°C, 2 h) 0.7‐μm GF/F glass fibre filter to quantify chlorophyll *a* (Chl*a*, a proxy for phytoplankton biomass). GF/F filters were placed in aluminium foil and stored on ice in the dark. Water filtrate passing through the GF/F was collected into triple‐rinsed, pre‐combusted glass scintillation vials for dissolved organic carbon (DOC), total dissolved nitrogen (TDN) and total dissolved phosphorus (TDP) analysis and kept on ice. Free‐living microbes were collected into pre‐sterilised carboys (10% bleach) and rinsed several times with < 64‐μm lake water on‐site. We filtered lake water using a hand syringe and tubing (10%‐bleach sterilised), collecting microbes onto a 0.22‐μm Sterivex filter cartridge (Millipore) until filters were loaded with material (300–1080 mL). Loaded Sterivex filters were capped and placed into Whirlpaks and stored on ice in the field. All samples were transported to the laboratory where they were stored (−20°C [except Sterivex, −80°C]). DOC samples were acidified with HCl (pH of < 2) and stored at room temperature.

We sampled zooplankton communities in each lake using a 64‐μm mesh (30.5 cm diameter) plankton net. Plankton were collected using a single vertical tow from 1 m above the deepest recorded depth of the lake. Zooplankton (> 64 μm) were preserved in 70% ethanol for later microscopic identification and enumeration. For microbiome analyses, we collected zooplankton using several vertical tows, concentrating the zooplankton community sample to > 250 μm size fraction and storing them in insulated thermoses filled with < 64‐μm filtered lake water and transported to SNARL.

### 2.3 Water Chemistry Analysis

We extracted Chl*a* by placing the GF/F filters individually into scintillation vials in 90% acetone, which were kept at 4°C and in the dark for 24 h. Chl*a* was quantified as μg/L concentration on a Turner Trilogy fluorometer (485/685 nm excitation–emission) calibrated against certified chlorophyll standards. DOC samples were analysed at the University of California Davis Analytical Laboratory on a Skalar Formacs TOC Analyzer with a detection limit of 0.5 mg/L using test method ASTM D5904‐02. TDN and TDP were measured on a Lachat QuikChem 8500 series 2 Flow‐Injection Analyzer using USGS I‐4650‐03 for external digestion for TN and US‐EPA method 365.3 for TP analysis (detection limit of 3.1 μg/L P) at the University of Hawai‘i at Hilo Analytical Laboratory.

### 2.4 Zooplankton Community Analysis

Zooplankton taxa (preserved in 70% ethanol) were identified to the lowest possible taxonomic levels (cladocerans to genus [*Daphnia*, *Ceriodaphnia, Bosmina, Chydorus, Holopedium* and *Polyphemus*], copepods [calanoid, cyclopoid and nauplii], and rotifers [*Keratella*, *Kellicotia* and *Asplanchna*]) and counted under a stereoscope (Leica Biosystems). We used a Folsom plankton splitter for sub‐sampling to count a proportion of the sample when communities were too dense to accurately count the full sample (> 1000 individuals per sample). Zooplankton densities were calculated as the number of organisms per litre of lake water, and the total density of all zooplankton was calculated as the sum of densities for all taxa.

### 2.5 DNA Extraction, PCR and 16S rRNA Gene Sequencing

Living zooplankton taxa were sorted by taxon (*Daphnia*, *Bosmina*, *Ceriodaphnia*, *Holopedium*, calanoid copepods and cyclopoid copepods) using a stereoscope (Leica Biosystems) and placed into 6‐well plates filled with distilled water (Eckert et al. 2021). Once separated, zooplankton remained in wells for ca. 30 min to aid in the removal of water‐associated bacterioplankton and gut clearance (Murtaugh 1985; Tsuda and Nemoto 1987); zooplankton were subsequently added to microcentrifuge tubes filled with 100% molecular grade ethanol and frozen (−80°C). To avoid cross‐contamination of zooplankton‐associated microbiomes from different lakes and sampling time points, all materials were rinsed in 100% molecular grade ethanol and utensils flame sterilised. Initial trials of 16S rRNA gene sequencing showed that there was no difference in species richness of microbes associated with *Daphnia* or calanoid copepods when grouped in 10–30 or 30–50 individuals, respectively (data not shown). Therefore, the number of individual zooplankton in each DNA extraction tube (*n* = 30 *Daphnia*, *n* = 50 *Bosmina*, *n* = 50 *Ceriodaphnia*, *n* = 10 *Holopedium, n* = 50 calanoid copepod and *n* = 50 cyclopoid copepod) was selected to optimise biomass for DNA extraction while allowing for sufficient within‐site‐and‐time technical replicates (*n* = 5 replicates per host where possible). Zooplankton samples with fewer (*n* = 8, with 8–20 individuals) and greater (*n* = 4, with ca. 100 individuals) numbers than those listed above were also included in DNA extraction to evaluate whether beta diversity was affected by the number of individuals used in extraction. Zooplankton microbiomes include the whole body (corpus) of multiple pooled individuals and therefore represent both the internal and external bacterial communities associated with crustaceans. In total, 347 zooplankton samples were processed and stored at −80°C until DNA extraction.

We used the Qiagen DNeasy Powersoil Pro Kit to extract DNA from the zooplankton and their associated microbiomes, following the manufacturer's protocol, eluting zooplankton DNA into 75 μL of elution buffer. We extracted DNA from the water filters using the Qiagen DNeasy PowerWater Sterivex Kit, following the manufacturer's protocol. We included negative controls (new power bead tubes [*n* = 16] and Sterivex filters [*n* = 2]) for both types of DNA extraction. Pre‐PCR DNA concentrations were quantified through fluorometric quantification using the Qubit 1× dsDNA high‐sensitivity kit (Thermo Fisher Scientific). All extracted DNA and controls (*n* = 368) were stored at −20°C until sequenced.

Approximately 290 bp of the V4 region of the prokaryotic 16S rRNA gene from bacteria and archaea was amplified in each sample using polymerase chain reaction (PCR). In each well of a 96‐well PCR plate, we added 2.5 μL of DNA, 5 μL of forward primer 515F: 5′ GTGYCAGCMGCCGCGGTAA‐3′, 5 μL of reverse primer 806R: 5′‐GGACTACNVGGGTWTCTAAT‐3′ and 12.5 μL KAPA HiFi HotStart ReadyMix (Roche Diagnostics), for a total of 25 μL reaction volumes. The primers used in this reaction were developed by Caporaso and colleagues (2011) and later modified (Apprill et al. 2015; Parada et al. 2016) to improve primer degeneracy to reduce sequencing bias. Thermocycling was conducted at the following temperatures and durations: initial denaturation at 95°C for 3 min, and 30 cycles of denaturation at 95°C for 30 s, followed by annealing at 58°C for 15 s, extension at 72°C for 30 s and final extension at 72°C for 5 min. Two negative controls (DEPC‐treated water from Thermo Fisher Scientific) and one positive control (ZymoBIOMICS Microbial Community DNA Standard) were included per PCR plate. Gel electrophoresis was used to qualitatively verify PCR amplification in samples and positive controls; negative controls showed no amplification. We used a second PCR to anneal Illumina Nextera XT i7 and i5 adapters, and DNA samples were normalised and pooled using the Just‐a‐Plate 96 PCR Normalization and Purification Kit (Charm Biotech). Post‐PCR DNA concentrations were then quantified using the Qubit high sensitivity assay. Sequencing was performed on a 1 mL sub‐sample of the pooled library at the University of California Davis Genome Center where a final Pippin size‐selection step was performed before sequencing as a paired‐end 250 run on an Illumina Miseq platform using v2 chemistry in a single sequencing run.

### 2.6 Bioinformatic Pipeline

We used cutadapt v.4.3 to remove the primers and adapters from Illumina high‐throughput DNA sequencing fragments (fastq files). The trimmed gene sequences were then processed through the DADA2 pipeline (Callahan et al. 2016) in R (version 4.3.3) (R Core Team 2024). Forward and reverse read quality profiles were inspected, and forward reads truncated at 225 (forward) 200 (reverse) positions. Following sequence processing, taxonomy was assigned to amplicon sequence variants (ASV) using the 2021 SILVA 138.1 reference database for 16S rRNA gene sequences (Quast et al. 2013). DNA extraction controls were used to identify and remove any contaminant ASVs from the dataset, using the decontam package v.1.16.0 (Davis et al. 2018). Microbial communities were analysed using phyloseq v.1.42.0 (McMurdie and Holmes 2013). ASVs not found in > 1 sample and those assigned as eukaryotes, mitochondria or chloroplasts were removed. Biological samples yielding < 100 gene sequences were also removed. In total, 5,007,570 sequences across 328 samples passed quality control (Figure S2). Due to low sequencing depth and replication, *Bosmina* and *Ceriodaphnia* samples were removed. The remaining gene sequences were rarified to an even sequencing depth (500 reads per sample) (see Supporting Information), and a final 16S library of 140,500 sequences representing 1959 ASVs across 281 samples (*n* = 253 zooplankton, *n* = 28 water) was used in downstream analysis.

### 2.7 Statistical Analyses

All statistical analyses were performed using R version 4.3.3 (R Core Team 2024). Linear models were used to test differences in the total zooplankton and taxa‐specific densities among sampling time points and lakes. Analysis of variance (ANOVA) tables were generated using type‐II sum of squares in the *car* package (Fox and Weisberg 2019). Assumptions of ANOVA were confirmed by graphical inspection of model residuals and quantile–quantile plots, with log‐transformations applied where assumptions were not met. Post hoc contrasts were calculated using estimated marginal means in *emmeans* (Lenth et al. 2021).

We examined bacterial alpha (*α*) diversity using the Shannon diversity index and observed richness (i.e., ASV richness, rarefied to 500 reads per sample) calculated in ‘estimate_richness’ in *phyloseq* (McMurdie and Holmes 2013). We tested the effects of sampling time points (*n* = 5), lakes (*n* = 6) and differences among sample types (four zooplankton taxa and water; *n* = 5 groups) on Shannon diversity and observed richness using linear models of main effects, with ANOVA tables generated using type‐II sum of squares and post hoc contrasts calculated in *emmeans*. Due to an unbalanced design, interaction terms were excluded.

We visualised spatiotemporal variation in free‐living and zooplankton‐associated microbiomes (beta [β] diversity) across sampling time points, lakes and sample types/taxa (zooplankton and water) using non‐metric multidimensional scaling (NMDS) analysis on a Bray–Curtis dissimilarity matrix (‘metaMDS’ in *vegan* [Oksanen et al. 2022], *k* = 3 dimensions). Euclidean distance was calculated among NMDS centroids for sampled types as a metric for variance in bacterial beta diversity. We then used principal coordinate analysis (PCoA) on distance matrices calculated with the ‘distance’ function in *phyloseq* (McMurdie and Holmes 2013) to test differences among free‐living and zooplankton microbiomes using three distance matrices that account for abundance and compositional changes (Bray–Curtis), as well as phylogenetic differences among taxa (UniFrac [Lozupone and Knight 2005]) unweighted and weighted by read abundance (Lozupone et al. 2007). We used permutational multivariate analysis of variance (PERMANOVA) to test effects of time points, lakes and sample types on microbial beta diversity for each ordination approach (Bray–Curtis, and weighted and unweighted Unifrac distance) with 999 permutations in ‘adonis2’ (Oksanen et al. 2022). Homogeneity of group dispersion was evaluated using ‘betadisper’ in *vegan*, with differences among groups determined from post hoc comparisons with Holm adjusted *p*‐values using ‘pairwise.adonis2’ in *pairwiseAdonis* (Martinez Arbizu 2020).

We evaluated zooplankton taxa‐specific beta diversity by subsetting communities within sample types (*Daphnia*, calanoid copepods, cyclopoid copepods and water) and used Bray–Curtis dissimilarity and NMDS to plot environmental data with significant correlations with NMDS axes using ‘envfit’ in *vegan*. *Holopedium* was excluded due to low replication. We used PERMANOVA of Bray–Curtis dissimilarity to test spatiotemporal effects (time, lake‐basins) at higher grouping levels: order Cladocera (*Daphnia*, *Holopedium*), class Copepoda (calanoids, cyclopoids) and water‐bacterioplankton. Finally, we used relative abundances of bacterial classes to visualise differences among Cladocera, Copepoda and water/bacterioplankton samples. Bacterial classes at < 5% relative abundance were grouped as ‘other’.

Spatial and environmental effects on the zooplankton communities and microbial communities (free‐living and host‐associated) were examined using stepwise selection (‘stepAIC’) in the package *MASS* (Venables and Ripley 2002). The correlation between variables was first inspected using a correlation matrix with *ggcorplot* (Kassambara 2022), and TDN and TDP were excluded due to an unbalanced matrix. We then used global linear models including spatial (latitude, elevation) and environmental variables (water temperature, pH, conductivity, DOC, Chl*a* and dissolved oxygen [DO]) using the following metrics as response variables: total zooplankton density and zooplankton community Shannon diversity, microbial community Shannon diversity (free‐living, zooplankton‐associated) and the relative abundance of the four most common bacteria classes across bacterioplankton and zooplankton. PERMANOVA was used to contrast spatial and environmental predictors of beta diversity in bacterioplankton and zooplankton microbiomes. We used ‘bioenv’ in *vegan* with scaled spatial environmental data to determine the metrics with highest rank correlation with free‐living microbial communities or zooplankton microbiomes, collectively (all hosts) and among host taxa.

## 3 Results

### 3.1 Lake Environmental Metrics

**FIGURE 2:**
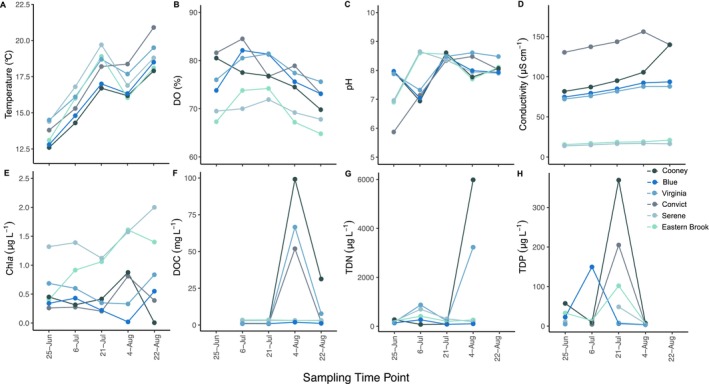
Environmental and water chemistry metrics measured in each of the six lakes across five sampling time points: (A) lake surface temperature, (B) dissolved oxygen (DO), (C) pH, (D) conductivity, (E) chlorophyll *a* (Chl*a*), (F) dissolved organic carbon (DOC), (G) total dissolved nitrogen (TDN) and (H) total dissolved phosphorus (TDP). Missing points represent samples that were lost or excluded from analyses.

Environmental conditions varied among sampling time points and lakes (Figure 2), with lakes in the same basin and in close proximity being more similar to each other (Figure 1). Lake water temperatures increased over the summer season from 12.6°C to 20.9°C, while dissolved oxygen (DO%) decreased as waters warmed. The two shallow lakes in the southern basin (Eastern Brook and Serene) had consistently lower DO% and conductivity but higher Chl*a* concentrations (indicating higher primary productivity) compared to other lakes (Figure 2). Frequent rain events (31 July through 9 August, Figure S1) caused pulsed increases in DOC and nutrients (TDN, TDP) throughout the study period, which were most evident in Cooney, Virginia and Convict Lakes. With the exception of rain events, DOC was low across all lakes and time points (< 8 mg/L) (Figure 2).

### 3.2 Zooplankton Community Composition and Density

**FIGURE 3:**
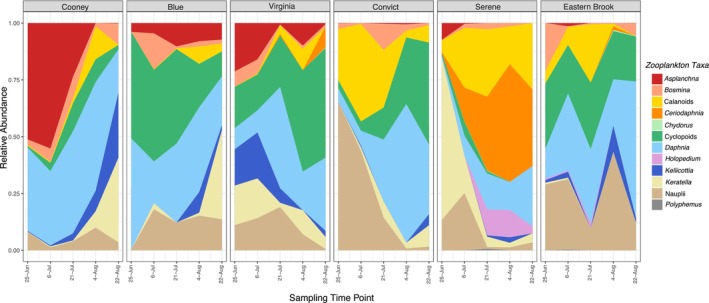
Relative abundances of zooplankton taxa (as proportion of total density per litre) in communities of six mountain lakes in California's Eastern Sierra Nevada across five sampling time points.

The relative abundance of zooplankton taxa differed across lakes (Figure 3). Communities were dominated by *Daphnia*, followed by cyclopoid copepods, calanoid copepods and nauplii, which tended to be more abundant early in the summer season (Figure S3). *Asplanchna* rotifer densities and relative abundance were dominant in plankton communities of northern lakes (Cooney, Blue and Virginia) (Figures 3 and S3), while *Keratella* rotifers abundance varied among lakes across the sampling season (Figure 3). Zooplankton Shannon diversity did not differ among time points or lakes (*p* ≥ 0.425) (Table S1) but showed a positive correlation with temperature (*p* = 0.047) (Table S2). Total zooplankton density (number of individuals L^−1^) changed through time (but not among lakes) (*p* < 0.001) (Table S1, Figure S3) and decreased through the season as lake temperatures increased (*p* = 0.008) (Table S2).

### 3.3 Microbiome Alpha Diversity

**FIGURE 4:**
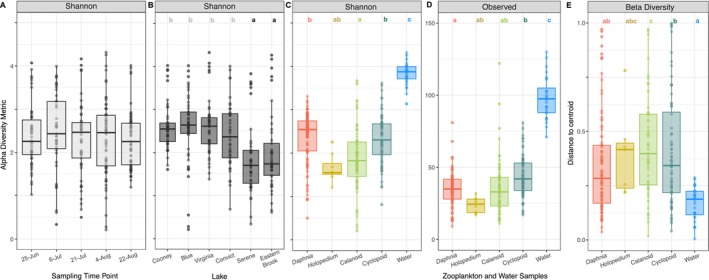
(A–D) Shannon diversity of bacterial communities across (A) sampling time points, (B) lakes and (C) among zooplankton and water samples. (D) Observed ASV richness by sample type (zooplankton and water). (E) Beta diversity as average distance to group centroids for two axes of non‐metric multidimensional scaling ordination (NMDS1‐2). Each point represents a microbial community. Box plots depict the median (bold centre line), first and third quartiles (lower and upper bounds) and whiskers (1.5× the distance between first and third quartiles). Letters indicate significant post hoc effects and pairwise differences (*p* < 0.05).

Shannon diversity and observed richness of bacterial communities (grouped across all sample types) did not differ across sampling time points (*p* ≥ 0.175) but were different among lakes (*p* < 0.001) and sample types (*p* < 0.001) (Table 2). Shannon diversity was lowest in the southernmost lakes (Serene and Eastern Brook) compared to the other four lakes (Figure 4B). Additionally, Shannon diversity and observed richness were higher in water samples compared to zooplankton (Figure 4C,D). Zooplankton microbiomes Shannon diversity was highest in cyclopoids and *Daphnia* (median: 2.5–3.9); observed richness was highest in copepods and was similar among other groups (median: 35–42 ASVs) except for *Holopedium* (median: 25 ASVs).

**TABLE 2:** Analysis of variance tables testing the effects of sampling time point, lakes and sample types (four zooplankton taxa and water) on bacterial community alpha diversity (Shannon diversity, observed richness).

	df	SS	*F*‐value	*Pr (> F)*
*Shannon diversity*				
Time point	4	0.687	0.604	0.660
Lake	5	18.559	13.059	**< 0.001**
Sample types	4	76.273	67.0835	**< 0.001**
Residuals	267	75.894		
*Observed richness*				
Time point	4	1246	1.598	0.175
Lake	5	6868	7.044	**< 0.001**
Sample types	4	98,071	125.737	**< 0.001**
Residuals	267	52,063		

*Note:* Significant effects (*p* < 0.05) are in bold. Tables generated with Type‐II SS from the *car* package. Models represent best fits from AIC model selection.

Abbreviations: df, degrees of freedom; SS, sum of squares.

**FIGURE 5:**
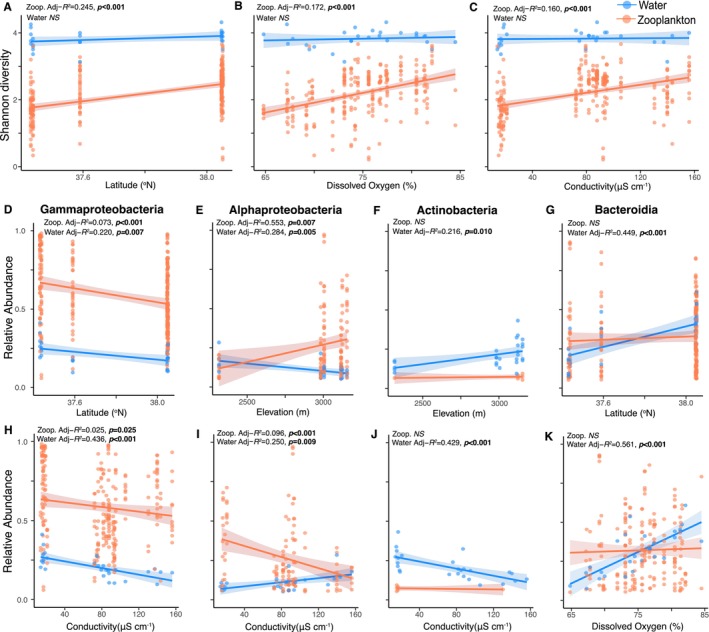
Plots of spatial and environmental variables with strongest correlations with microbial communities in water bacterioplankton and zooplankton hosts (see Table S6). (A–C) Correlations between Shannon diversity (*top row*) and latitude, dissolved oxygen and conductivity. (D–K) Correlations between the relative abundance of bacteria classes (Gammaproteobacteria, Alphaproteobacteria, Actinobacteria and Bacteroidia) and spatial (*middle row*) and environmental predictors (*bottom row*). Adjusted‐*R* ^2^ and *p*‐values are derived from one‐way linear models. *NS* represents a non‐significant model fit (*p* > 0.05).

Bacterioplankton Shannon diversity was not significantly affected by spatial or environmental conditions, whereas zooplankton Shannon diversity was best explained by a model containing a mix of spatial and environmental traits (Table S3). Testing these predictors in one‐way linear models, we found Shannon diversity in zooplankton microbiomes (but not bacterioplankton) increased with latitude, DO and conductivity (*p* < 0.001) (Figure 5A–C, Table S4).

### 3.4 Relative Abundance of Bacteria Taxa

**FIGURE 6:**
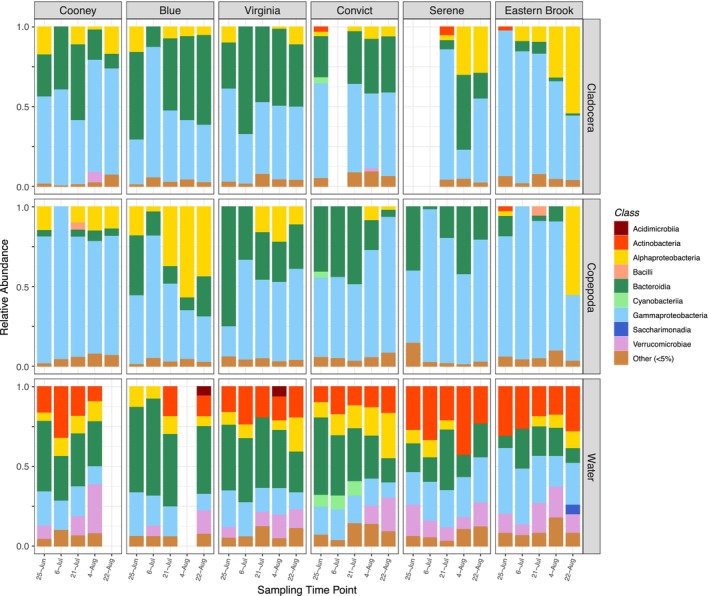
Relative abundance of bacteria classes among Cladocera (*Daphnia*, *Holopedium*) and Copepoda (calanoid and cyclopoid copepods) zooplankton and water in six lakes of the Eastern Sierra Nevada mountains at five time points across the summer season. Gaps in the data represent either absence of samples in a lake or time or the loss of samples due to low sequencing depth.

The relative abundances of bacteria classes in zooplankton microbiomes and bacterioplankton varied through time within each lake (Figure 6). In zooplankton, the dominant classes of bacteria (averaged across lakes and times in each plankton host (*n* = 4 hosts)) were Gammaproteobacteria (42%–67%), Bacteroidia (19%–54%) and Alphaproteobacteria (11%–16%). Overall, Gammaproteobacteria was most abundant across all locations and sampling points in both cladocerans and copepods, while Bacteroidia and Alphaproteobacteria abundance in hosts varied regionally (north vs. south) and among hosts. The free‐living bacterioplankton were less variable through time, with Bacteroidia (31%), Actinobacteria and Gammaproteobacteria (20%–21% each), Verrucomicrobiae and Alphaproteobacteria (10%–11% each) being most common.

At the family level (pooled across time points and lakes), zooplankton showed high abundances of Comamonadaceae (Gammaproteobacteria, 14%–40%) and Flavobacteriaceae (Bacteroidia, 8%–23%) (Figure S4). *Holopedium* displayed unique associations and was dominated by Chitinophagaceae (Bacteroidia, 24%) and Wellsellaceae (Bacteroidia, 20%). Water bacterioplankton families showed high abundance of novel taxa, including Sporichthyaceae (Actinobacteria, 19%), as well as many of the bacterial families found in zooplankton (Comamonadaceae, Flavobacteriaceae and Chitinibacteraceae) [7%–11%], albeit 50% of the bacterioplankton consisted of families at low abundance (< 5%) (Figure S4).

Stepwise model selection showed the relative abundance of the most prevalent bacteria classes (Gammaproteobacteria, Alphaproteobacteria, Actinobacteria and Bacteroidia) in free‐living bacterioplankton and zooplankton microbiomes was most often influenced by latitude, conductivity and DO% (Table S5, Figure 5D–K). The significance and direction of these effects, however, differed between water and zooplankton samples. In univariate models (Table S6), Gammaproteobacteria relative abundance declined with latitude and conductivity in both bacterioplankton and zooplankton (Figure 5D,H). In bacterioplankton, Alphaproteobacteria declined with elevation and increased with conductivity, while the opposite pattern was observed in zooplankton (Figure 5E,I). Actinobacteria relative abundance increased with elevation and declined with conductivity in bacterioplankton alone (Figure 5F,J). Bacteroidia relative abundance significantly increased with latitude and DO% in bacterioplankton alone (Figure 5G,K) (Table S6).

### 3.5 Microbiome Beta Diversity

**FIGURE 7:**
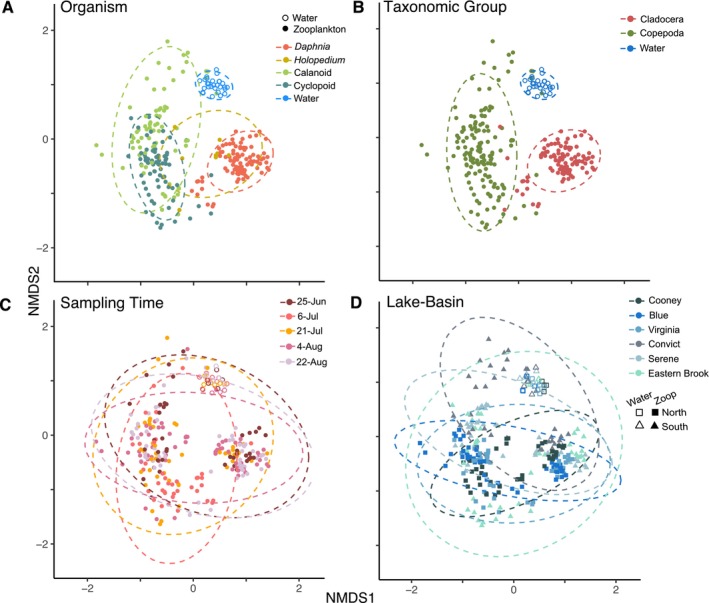
Bacterial communities associated with zooplankton and in water of six mountain lakes across five sampling time points. Non‐metric multidimensional scaling (NMDS) ordination plots grouped by (A) host organism (species or functional group), (B) taxonomic group, (C) sampling time points and (D) across lake‐basins, being in the north (Cooney, Blue and Virginia) or the south (Convict, Serene and Eastern Brook). Each point is a separate microbial community sample. NMDS stress = 0.163.

We used NMDS ordination to test for spatiotemporal and hierarchical effects in beta diversity related to time point, lakes, sample types and higher taxonomic grouping (Figure 7). PERMANOVA showed significant differences in Bray–Curtis dissimilarity attributed to time point, lakes and sample types and their interactions, with sample type (bacterioplankton vs. zooplankton) explaining the greatest variation (*R* ^2^ = 0.277, *p* = 0.001) (Table 3), followed by lake (*R* ^2^ = 0.132, *p* = 0.001) and lake‐by‐sample type interaction (*R* ^2^ = 0.164, *p* = 0.001), with minimal variance explained by time points (*R* ^2^ = 0.061, *p* = 0.001). All five sample types (four zooplankton taxa and water) were different from each other in post hoc comparisons (Holm adjusted‐*p* = 0.010). Free‐living bacterial communities in water were distinct and less variable than those associated with zooplankton hosts. Phylogenetic distance metrics (Unifrac and weighted Unifrac) produced similar clustering by sample type, with *Daphnia*‐associated microbiomes distinct from other zooplankton (Figure S5; Table 3).

**TABLE 3:** PERMANOVA results testing the effects of sampling time points, lakes and community sample types (four zooplankton groups [cladocerans: *Daphnia*, *Holopedium;* copepods: calanoids, cyclopoids], water) on microbial communities using Bray–Curtis dissimilarity, unweighted and weighted Unifrac distance.

	df	SS	*R* ^2^	*F*	*Pr* (> *F*)
*Bray–Curtis dissimilarity*					
Time point	4	7.163	0.061	26.522	**0.001**
Lake	5	15.527	0.132	45.990	**0.001**
Sample type	4	32.520	0.277	120.404	**0.001**
Time point: Lake	20	13.550	0.116	10.034	**0.001**
Time point: Sample type	14	7.086	0.060	7.496	**0.001**
Lake: Sample type	15	19.193	0.164	18.950	**0.001**
Time point: Lake: Sample type	39	10.129	0.086	3.846	**0.001**
Residual	179	12.087	0.103		
Total	280	117.256	1.000		
*Weighted Unifrac distance*					
Time point	4	4.657	0.069	28.163	**0.001**
Lake	5	8.915	0.133	43.129	**0.001**
Sample type	4	18.264	0.272	110.446	**0.001**
Time point: Lake	20	7.072	0.105	8.553	**0.001**
Time point: Sample type	14	4.242	0.063	7.330	**0.001**
Lake: sample type	15	10.569	0.158	17.044	**0.001**
Time point: Lake: Sample type	39	5.978	0.089	3.708	**0.001**
Residual	179	7.400	0.110		
Total	280	67.098	1.000		
*Unweighted Unifrac distance*					
Time point	4	4.247	0.053	7.686	**0.001**
Lake	5	9.524	0.118	13.788	**0.001**
Sample type	4	13.612	0.168	24.632	**0.001**
Time point: Lake	20	8.597	0.106	3.111	**0.001**
Time point: Sample type	14	3.807	0.047	1.968	**0.001**
Lake: Sample type	15	9.021	0.112	4.353	**0.001**
Time point: Lake: Sample type	39	7.371	0.091	1.368	**0.001**
Residual	179	24.730	0.306		
Total	280	80.910	1.000		

*Note:* Significant effects (*p* < 0.05) are in bold.

Abbreviations: df, degrees of freedom; SS, sum of squares.

The distances to NMDS1 and NMDS2 centroids were shorter in free‐living compared to zooplankton‐associated microbiomes, indicating lower beta diversity (Figure 4E). *Daphnia* were most distinct from other taxa (Figure 7A) and cladocerans (*Daphnia* and *Holopedium*) together were distinct from copepods (Figure 7B) which showed high overlap at lower taxonomic levels (cyclopoids, calanoids) and greater dispersion among samples (Figure 4E). Sampling time points and lakes each showed high degrees of overlap and were poorly distinguished in NMDS ordination, with water samples clustering relative to zooplankton (Figure 7C,D).

Within factor levels (time points, lake location or sample types), Bray–Curtis beta dispersion showed significant differences in beta dispersion among time points (*p* = 0.037) and sample types (*p* < 0.001) but not lakes (*p* = 0.060). A PERMANOVA of spatial and environmental variables (Table S7) showed water bacterioplankton beta diversity was most affected by latitude (*R* ^2^ = 0.186, *p* = 0.001) and elevation (*R* ^2^ = 0.166, *p* = 0.001). Conversely, beta diversity of zooplankton microbiomes (across all hosts) was less explained by spatial (latitude and elevation, *R* ^2^ = 0.040–0.060, *p* = 0.001) and environmental parameters (DO% and conductivity, *R* ^2^ = 0.014–0.019, *p* = 0.001).

**FIGURE 8:**
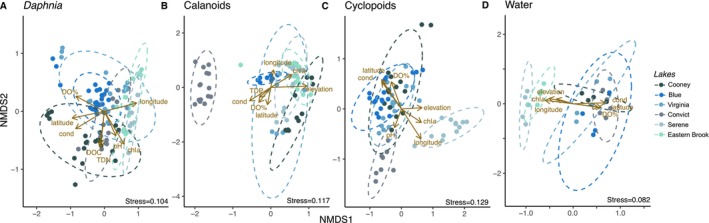
Non‐metric multidimensional scaling (NMDS) ordination on Bray–Curtis dissimilarity matrix of bacterial communities associated with (A) *Daphnia*, (B) calanoid and (C) cyclopoid copepod zooplankton, or in (D) water across six lakes and five sampling time points. Vectors represent environmental conditions that significantly correlate (*p* < 0.05) with NMDS axes. Ellipses represent 95% confidence intervals.

We subset NMDS ordination among the three dominant zooplankton groups (*Daphnia*, cyclopoid copepods and cyclopoid copepods) and water to examine environmental and spatial patterns affecting beta diversity in each sample type/host taxa. We again found a greater influence of lake environment (*R* ^2^ = 0.356–0.0520, *p* = 0.001) on beta diversity for zooplankton and water compared to time points (*R* ^2^ = 0.131–0.180, *p* = 0.001) (Figure 8, Table 4). Within zooplankton host taxa or water‐bacterioplankton, beta dispersion was not different among time points (*p* ≥ 0.240) or lakes for most samples (*p* ≥ 0.490) but did vary among lakes for calanoids (*p* = 0.004). In post hoc contrasts, zooplankton microbiome composition differed from lake to lake for each zooplankton group (Holm adjusted‐*p* ≤ 0.015); however, bacterioplankton did not (*p* ≥ 0.060), although Serene and Eastern Brook formed a distinct cluster away from other lakes (Figure 8D). Bioenv models showed zooplankton microbiome beta diversity was most strongly correlated with pH + conductivity (*Daphnia, r* = 0.49), latitude + elevation (calanoids, *r* = 0.74) and latitude + pH + conductivity (cyclopoids, *r* = 0.71); bacterioplankton most strongly correlated with latitude + conductivity (*r* = 0.78).

**TABLE 4:** PERMANOVA tables testing the effects of intra‐seasonal (sampling times) and spatial effects (lake habitats) on *Daphnia*, calanoid and cyclopoid microbiomes and free‐living (water) microbial community beta diversity using Bray–Curtis dissimilarity.

	df	SS	*R* ^2^	*F*‐value	*Pr* (> *F*)
*Daphnia*					
Time point	4	4.127	0.180	23.622	**0.001**
Lake	5	8.168	0.356	37.399	**0.001**
Time point: Lake	17	7.517	0.328	10.123	**0.001**
Residual	71	3.101	0.135		
Total	97	22.914	1.000		
*Calanoid*					
Time point	4	4.252	0.159	12.499	**0.001**
Lake	5	12.142	0.455	28.556	**0.001**
Time point: Lake	12	5.866	0.220	5.748	**0.001**
Residual	52	4.422	0.166		
Total	73	26.681	1.000		
*Cyclopoid*					
Time point	4	3.976	0.178	11.717	**0.001**
Lake	4	9.230	0.413	27.202	**0.001**
Time point: Lake	12	4.734	0.212	4.651	**0.001**
Residual	52	4.411	0.198		
Total	72	22.351	1.000		
**Water**					
Time point	4	0.991	0.131	1.696	**0.012**
Lake	5	3.924	0.520	5.374	**0.001**
Residual	18	2.629	0.349		
Total	27	7.543	1.000		

*Note:* Significant effects (*p* < 0.05) are in bold.

Abbreviations: df, degrees of freedom; SS, sum of squares.

## 4 Discussion

Our survey of six mountain lakes throughout the summer season (25 June–23 August) found distinct bacterial taxa in and on different crustacean zooplankton host species and in the lake epilimnion. We found consistent, scale‐dependent patterns of diversity between free‐living and host‐associated communities. Free‐living microbial communities (i.e., bacterioplankton) possessed greater alpha diversity at local (within lake) scales, while zooplankton‐associated microbes exhibited greater compositional (beta diversity) changes across space and time. These contrasts suggest differences in the importance of processes regulating community assembly through colonisation and drift for free‐living microbes relative to those associated with invertebrate hosts. We also found taxonomically diverse zooplankton hosts selected for distinct microbial associations, with copepods displaying greater microbiome variability among lake populations relative to cladocerans. Overall, *Daphnia* microbiomes (and cladocerans as a whole) were distinct from those of copepods, suggesting that unique ecological or physiological traits lead to divergence in microbiomes (Callens et al. 2016; Pfenning‐Butterworth et al. 2022). The symbiotic relationships between zooplankton and their associated bacteria are highly flexible, complex and dependent on many factors, making it difficult to attribute the contrast in cladoceran and copepod microbiomes to any single effect (Akbar et al. 2022; Eckert et al. 2021; Hegg et al. 2021). Nevertheless, the influence of host taxonomy on zooplankton microbiomes highlights the principal importance of zooplankton host selection in microbiome assembly in alpine lakes.

Higher alpha diversity and lower beta diversity (and beta dispersion) in bacterioplankton may be driven by fundamental differences in environmental versus organism‐associated habitats. For instance, large environmental reservoirs—such as lakes—may harbour greater microbial diversity relative to comparatively small host‐associated habitats (Nemergut et al. 2013). It is equally possible that fewer microbes are adapted to live in association with living hosts, indicating niche diversification across microbes (Samad et al. 2020). Bacterioplankton species may also accumulate in downstream populations relative to headwaters (i.e., stochastic mass effect processes) through unidirectional advection through watersheds, thereby reducing species turnover and increasing similarity among communities (Aguilar and Sommaruga 2020; Nelson et al. 2009; Shmida and Wilson 1985). Indeed, the relative significance of mass effects on bacterioplankton community composition has been shown to increase in oligotrophic lakes (such as those in the current study) relative to more productive systems (Van der Gucht et al. 2007). While speculative, these mechanisms may help explain the high prevalence of low‐abundance taxa (and lower beta diversity) in bacterioplankton relative to zooplankton microbiomes (Figures 6 and 7).

Overall, lower alpha and higher beta diversity in zooplankton hosts suggest that some combination of stronger host selection and reduced dispersal between hosts (horizontal transmission) structures zooplankton microbiomes compared to free‐living bacterioplankton. Interestingly, lakes consistently explained more variation in beta diversity than time (Table 3), even though the environmental conditions varied as much or more through time than among lakes. These patterns suggest that, despite considerable seasonal variation within lakes, dispersal limitation across space has a stronger effect on community turnover than temporal variation within sites. Thus, microbial communities are more effectively sorted across space than time, even though seasonal environmental variation is comparable to or greater than spatial gradients for many variables (e.g., temperature, Figure 2).

### 4.1 Temporal Effects on Microbial Communities

In alpine lakes, summer marks a brief period when air and water temperatures increase, with melting of snow transporting nutrients into lakes that support phytoplankton (Zohary et al. 2021) and zooplankton growth (Heinle 1969). Annual changes in phytoplankton and zooplankton community succession can influence both the richness and abundance of bacterioplankton (Newton et al. 2006), establishing trophic interactions as an important factor driving bacterioplankton communities (Kent et al. 2004). In our sampling (25 June–23 August), changes in zooplankton communities, their densities or lake productivity did not correspond with shifts in bacterioplankton communities. Instead, we found bacterioplankton community composition and diversity to be highly consistent across the summer season compared to zooplankton‐associated microbiomes, although spatial structure was evident in both groups (Figure 8). Moreover, while progressive warming in lakes (ca. +5°C–7°C) coincided with declines in zooplankton densities, temperature alone was a poor predictor of free‐living and zooplankton microbial community diversity and composition. In other studies, changes in water temperature (Lindström et al. 2005) and lake phenologies were found to be important drivers of bacterioplankton (Shade et al. 2007; Sommer et al. 2012) and zooplankton microbiomes composition (Frankel‐Bricker et al. 2020; Hegg et al. 2021). An interseasonal study of an alpine lake in China revealed bacterioplankton alpha diversity differed across seasons, being higher in winter than in summer (Shang et al. 2022). Seasonal cycles of bacteria in alpine lakes are likely influenced by drastic temperature shifts that occur at high elevations among seasons. Thus, it is possible that lakes in our study were at comparatively similar phenologies for the summer season, resulting in reduced significance of within‐season effects on microbial communities relative to those observed among seasons (i.e., within a year) or across summers through time (i.e., among years). However, interseasonal differences of zooplankton‐associated microbial communities and how they may be influenced by seasonal changes in environmental conditions or interactions with seasonally shifting bacterioplankton communities are not well understood. Future studies may therefore benefit from quantifying changes in bacterioplankton, phytoplankton and zooplankton (and their microbiomes) at larger temporal scales to better predict effects of environment, succession and their feedback across time (Phillips et al. 2021; Yannarell and Triplett 2004).

### 4.2 Spatial Effects on Microbial Communities

Variance in microbial communities was most explained by sample type (bacterioplankton vs. zooplankton), followed by lake and the interaction of sample type and lake. These results indicate deterministic factors varying among lakes (e.g., environmental species sorting) interacted with host mechanisms (e.g., biological filtering) to shape bacterial community composition at different scales. Across space, lakes that were closer geographically tended to have more similar environmental conditions (in particular Chl*a*, conductivity and DO), as well as similar bacterioplankton and zooplankton microbiomes (Figures 2 and 6). For instance, we observed greater relative abundance of Bacteriodia and Alphaproteobacteria (northern lakes) and Gammaproteobacteria and Actinobacteria (southern lakes). Alpha diversity (free‐living and host‐associated) was also lower in southern lakes (Eastern Brook, Serene), and free‐living bacterioplankton beta diversity partitioned between north and south basins (Figures 4B and 8D). However, the strength of spatial effects differed among bacterioplankton and zooplankton microbiomes. For instance, latitude and elevation accounted for 35% of variance in bacterioplankton beta diversity, but only 10% for zooplankton (Table S7). In contrast, zooplankton microbiome Shannon diversity positively correlated with latitude, whereas bacterioplankton Shannon diversity did not (Figure 5A). Ultimately, our results reveal contrasting roles of geospatial and environmental factors in shaping the diversity and composition of free‐living and zooplankton‐associated microbial communities. The greater proportion of variance explained by spatial and environmental factors in free‐living bacterioplankton relative to zooplankton microbiomes (57% vs. 18%, respectively) indicates a greater influence of spatial and physicochemical factors on bacterioplankton, possibly driven by a combination of proximity, dispersal limitations and environmental selection driving species sorting (Leibold et al. 2004; Yannarell and Triplett 2004). Nevertheless, lakes explained 36%–46% of variance in within‐zooplankton taxa microbiomes. Thus, while the assembly and maintenance of zooplankton microbiomes is fundamentally driven by host identity (Figure 7A), within‐host spatial structure (Figure 8) shows lake habitat—either through proximity or environmental filtering—is a key determinant of zooplankton microbiomes in alpine lakes.

### 4.3 Environmental Drivers of Bacterioplankton Abundance and Community Change

Differences in conductivity and dissolved oxygen among lakes, rather than seasonal (e.g., temperature) or punctuated nutrient perturbations (e.g., storm runoff)—were most important in structuring microbial communities across our summer sampling (Tables S3 and S4, Figures 5 and 7). Future studies would benefit from analysing the effects of temperature changes from winter to summer on both bacterioplankton and zooplankton microbiome structure and diversity. Bacterioplankton community diversity is responsive to changes in stratification, pH, temperature and carbon sources (Jones et al. 2009; Lindström et al. 2005; Schulhof et al. 2020; Shade et al. 2007, 2008), and in Sierra Nevada lakes, temperature and DOC quality strongly influence bacterioplankton community structure (Schulhof et al. 2020). With the exception of sporadic peaks in nutrients and DOC due to storms (Figures 2 and S1), DOC concentration was low during the summer season across lakes. The absence of clear DOC effects in our study may be an effect of consistent seasonal phenologies (Nelson 2009) and shared water chemistry properties in these systems across longer time periods (i.e., oligotrophic and algal‐dominated DOM pools) (Aguilar and Sommaruga 2020). Overall, the similarity in nutrient regimes and remote nature of alpine lakes in the Eastern Sierra (i.e., low urbanisation, agriculture impacts) likely reduced the significance of environmental selection observed in other studies that occur across lakes of contrasting nutrient regimes and degrees of human influence (Onana et al. 2025).

Conductivity and DO had strong effects on Shannon diversity and the relative abundance of the dominant bacterial taxa. Interestingly, DO and conductivity reflected local, within‐lake environmental conditions and the nestedness of lakes within basin/watershed instead of seasonal environmental change (Figure 2). For instance, zooplankton microbiome Shannon diversity positively correlated with DO% and conductivity, as well as latitude, while bacterioplankton showed no relationship with these predictors (Figure 5A–C). In contrast to Shannon diversity, the relative abundance of dominant bacteria classes in bacterioplankton and zooplankton was driven by a combination of spatial factors and environmental factors; however, the direction of these effects often differed among communities at different scales (Figure 5E–K). Gammaproteobacteria relative abundance universally declined with latitude and conductivity in bacterioplankton and zooplankton microbiomes. In contrast, bacterioplankton relative abundance was influenced by spatial patterns for Alphaproteobacteria (decreasing with elevation), Actinobacteria (increasing with elevation), and Bacteroidia (increasing with latitude), while these groups showed weak or no correlation in zooplankton microbiomes. Similarly, the relative abundance of all major bacteria classes in the bacterioplankton correlated with changes in conductivity, DO or both; in zooplankton, only Gammaproteobacteria and Alphaproteobacteria showed significant correlations with conductivity. These contrasting effects suggest spatial and environmental effects are stronger determinants of alpha diversity in zooplankton microbiomes, but not in bacterioplankton, which show greater stability among lakes and across environmental conditions. Instead, a combination of spatial and local environmental conditions has a greater influence on bacterioplankton community composition, acting to stimulate or suppress the abundance of prevalent bacteria taxa shared across lake environments.

The prevalence of bacteria classes in different habitats (free‐living, host‐associated) may also relate to niche diversification and their responsiveness (and tolerance) to changes in environmental conditions or resource availability. For instance, Actinobacteria are adapted to low‐nutrient environments and high UV (Warnecke et al. 2005), which may explain the abundance of Actinobacteria (family Sporichthyaceae) in the bacterioplankton of oligotrophic lakes and their absence in zooplankton microbiomes (Figures 6 and S4). Similarly, Alphaproteobacteria are competitive for chemically diverse nutrient substrates (Hutalle‐Schmelzer et al. 2010; Newton et al. 2011) and were dominant in bacterioplankton but highly variable in zooplankton hosts. Bacteroidia and Gammaproteobacteria—dominant in zooplankton microbiomes and bacterioplankton—are sensitive to resource availability and increase in response to algal blooms, phytoplankton community succession and high grazing pressures (Eiler and Bertilsson 2007; Newton et al. 2006; Šimek et al. 1999). Overall, these results show broad contrasts between bacterioplankton and zooplankton microbiomes expressed at high taxonomic levels of family and class, with greater biogeographical structure in bacterioplankton suggesting a stronger influence of environmental selection and dispersal limitations.

### 4.4 Contrasting Patterns in Crustacean Zooplankton Microbiomes

Microbiomes of cladocerans and copepods contained the same dominant classes of bacteria but in different relative abundances, which supports the horizontal transmission of microbes from the ambient water and the significance of host selection as mechanisms of microbiome assembly/composition in freshwater zooplankton (Figures 6 and 8) (Callens et al. 2020; Eckert et al. 2021; Mushegian et al. 2019). Members of Bacteroidia (family Flavobacteriaceae and Chitinophagaceae) and Gammaproteobacteria (family Comamonadaceae)—consisting of enteric taxa common in animal gut microbiomes as well as particle‐associated degraders of biopolymers (Eckert et al. 2021; Macke et al. 2017; Newton et al. 2011)—were dominant in both bacterioplankton and zooplankton. Other groups, namely Alphaproteobacteria (dominated by Chitinophagaceae and Candidatus Hepatincola) were highly variable in their host association and displayed both location and host‐specific patterns (Figures 6 and S4), possibly due to changes in nutrients, grazing pressure and diet (Pfenning‐Butterworth et al. 2022) (Figure 8) across the summer season (Hegg et al. 2021). As the microbiota associated with zooplankton (notably in their guts) can confer resistance to toxins (Macke et al. 2017) and support host performance under varying food availability (Callens et al. 2016), seasonal changes in freshwater zooplankton microbiomes may reflect dynamic associations to meet ecological challenges associated with fluctuations in resource availability and environmental conditions.

Within‐lake differences in environmental conditions and production also contributed to the separation of zooplankton microbiomes. While microbiomes of *Daphnia* populations were more homogeneous, microbiomes of copepods in Convict Lake (calanoids and cyclopoids) and Serene (cyclopoids) were distinct from other conspecific populations. These shifts in beta diversity positively (cyclopoids, Serene) and negatively (calanoids, Convict) correlated with Chl*a* and elevation and positively with pH (cyclopoids, Convict). The greater spatial separation and environmental influence on copepod‐associated bacteria, and zooplankton in general relative to free‐living bacterioplankton, may be due to host feeding behaviour acting to filter and facilitate microbiome assembly. For instance, while *Daphnia* microbiomes exhibit high plasticity (Callens et al. 2020; Eckert et al. 2021), they recruit and retain distinct microbial taxa in their guts from the microbial taxa that they are exposed to in the ambient water (Hegg et al. 2021). However, oligotrophic conditions of alpine lakes can favour greater bacterial attachment to hosts relative to eutrophic conditions (Grossart et al. 2009) and might have contributed to greater within‐host taxa clustering (Figures 5 and S4). The affinity for certain bacteria to be particle‐associated or found inside hosts may be important in our study, as we assessed whole‐corpus microbiome diversity (Eckert et al. 2021). Isolating gut tissues may reduce transient and/or epibiont taxa associated with external surfaces and food/prey items and provide greater resolution of zooplankton microbiomes (or tissue‐specific compartments hosting core and unique taxa) and their distinction from, and feedback to, the external bacterioplankton (Macke et al. 2020). Nevertheless, our analysis of whole organisms found zooplankton microbiomes differed among host taxa and displayed unique community structure and composition relative to bacterioplankton in water. Thus, while lineages of bacteria display a high degree of cosmopolitanism (Sommaruga and Casamayor 2009), local environmental factors and adaptations for oligotroph, copiotroph and host‐associated lifestyles are important in shaping bacterial community composition (Akbar et al. 2022; Newton and McLellan 2015).

## 5 Conclusion

Microbes and zooplankton are essential members of alpine lakes that serve many vital functions in nutrient cycling, water quality and trophic interactions. However, these sentinel ecosystems are threatened by rising global temperatures, decreasing snowpack and deteriorating water quality (Adrian et al. 2009; Pastorino et al. 2024), which may disrupt the complex relationships between free‐living and host‐associated microbial communities. Here, we find consistent sets of microbial taxa that were associated with the lake epilimnion and zooplankton hosts across lakes and sampling dates, suggesting that different host species select for (or are receptive to) particular symbionts. This result is consistent with the ‘core/satellite’ microbiome concepts (Neu et al. 2021), that some members of the hosts' microbiome vary in response to the environment while others are found consistently regardless of spatial or temporal environmental variation or the ecological context.

Although the free‐living bacterioplankton showed higher alpha diversity and more low‐abundance bacterial taxa (Figures 4 and 6), the microbiomes of all zooplankton hosts were more variable across space and time (Figures 7 and S5). This contrast suggests two possible mechanisms: either host‐associated microbiomes are more sensitive to environmental variation among lakes or sampling dates, or the homogenising effect of dispersal in space and time is more prevalent among environmental microbes. We cannot distinguish these two explanations in our data; however, some lines of evidence may point to the second. Many environmental microbes are absent or exceedingly rare in the zooplankton, suggesting a greater inventory of bacterial taxa in the environment that are either not adapted to symbiotic lifestyles or are not selectively retained by hosts. Host‐associated bacteria may also rely on host dispersal to move between lakes, while free‐living taxa may be more readily transported by wind or surface water advection (Nelson et al. 2009). The contrasting patterns of alpha and beta diversity in free‐living versus host‐associated microbes suggest that environmental selection and dispersal play different roles in the assembly of these two important communities.

## Author Contributions

C.B.W., M.G.P., J.B.S. and J.H.D. designed the study; M.G.P., E.M.D. and J.H.D. collected the data and performed laboratory analyses; C.B.W., M.G.P. and M.Y.D. analysed the data; all authors wrote the manuscript.

## Conflicts of Interest

The authors declare no conflicts of interest.

## Supporting information


**Data S1:** Supporting Information.

## Data Availability

All code and data are openly available on GitHub (https://github.com/cbwall/Zoops‐in‐Time) and archived at Zenodo (Wall 2025). Sequence data were submitted to the NCBI SRA at BioProject PRJNA1144600.
