# Supplementary material for: Geography and Host Identity Shape Intraseasonal Variation of Free‐Living and Zooplankton Associated Microbial Communities in Alpine Lakes
Source: Mol Ecol. 2025 Aug 12;34(19):e70069. doi: 10.1111/mec.70069 (PMC12456117; doi:10.1111/mec.70069)
Supplement: Supplementary file 1 — Data S1: Supporting Information. [file MEC-34-e70069-s001.pdf]

MOLECULAR ECOLOGY

Supplemental Information for:

Geography and host identity shape intraseasonal variation of free-living and zooplankton associated microbial communities in alpine lakes

Christopher B. Wall‡, Madeline G. Perreault‡, Margaret Y. Demmel, Evelyn M. Diaz, Joshua H. Dominguez, Jonathan B. Shurin

‡=both authors contributed equally and share first authorship

Table of Contents:

**Table S1.**	Page 2
**Table S2.**	Page 3
**Table S3.**	Page 4
**Table S4.**	Page 5
**Table S5.**	Page 6
**Table S6.**	Page 7
**Table S7.**	Page 8
**Figure S1.**	Page 9
**Figure S2.**	Page 10
**Figure S3.**	Page 11
**Figure S4.**	Page 12
**Figure S5.**	Page 13

MOLECULAR ECOLOGY

Table S1. Analysis of variance tables testing differences in zooplankton community Shannon diversity and zooplankton densities (number of individuals per liter) in samples collected in six mountain lakes across five sampling time points.

Zooplankton Shannon diversity	*df*	*SS*	*F-value*	*Pr(>F)*
Time Point	4	0.310	1.013	0.425
Lake	5	0.395	1.031	0.426
Residuals	20	1.533		
Total zooplankton density				
Time Point	4	6015	7.582	**<0.001**
Lake	5	1373	1.385	0.272
Residuals	20	3967		

df = degrees of freedom, *SS* = sum of squares, significant effects ($p<0.05$) are in bold. Tables generated with Type-II SS from the *car* package. Models represent best-fits from AIC model selection.

MOLECULAR ECOLOGY

Table S2. Analysis of variance tables testing environmental factors as predictors of zooplankton community alpha diversity (Shannon diversity) and total zooplankton densities in six mountain lakes across five sampling time points.

Zooplankton Shannon diversity	*df*	*SS*	*F-value*	*Pr(>F)*
Latitude	1	0.201	2.911	0.100
Conductivity	1	0.194	2.806	0.106
Temperature	1	0.299	4.337	**0.047**
Residuals	26	1.794		
Total zooplankton density				
Latitude	1	783.8	2.435	0.132
Elevation	1	636.9	1.978	0.172
Dissolved oxygen	1	731.1	2.271	0.145
Conductivity	1	619.1	1.923	0.178
Temperature	1	2278.1	7.076	**0.014**
Residuals	24	7726.7		

df = degrees of freedom, *SS* = sum of squares, significant effects (p<0.05) are in bold. Tables generated with Type-II SS from the *car* package. Models represent best-fits from AIC model selection.

MOLECULAR ECOLOGY

Table S3. Analysis of variance tables testing spatial and environmental variables as predictors of alpha diversity (Shannon diversity) in water-borne bacterioplankton and four zooplankton taxa collected in six mountain lakes across five sampling time points.

Diversity metric	*Sample type*	*Effect*	*df*	*SS*	*F-value*	*Pr(>F)*
**Shannon Diversity**	*Water*	Elevation	1	0.262	4.205	0.051
		Conductivity	1	0.200	3.221	0.085
		Residuals	25	1.556		
	Zooplankton	Latitude	1	9.264	31.218	**<0.001**
		log(Dissolved organic carbon)	1	1.823	6.142	**0.014**
		Chlorophyll	1	0.764	2.573	0.110
		pH	1	1.348	4.542	**0.034**
		Dissolved oxygen	1	2.134	7.191	**0.008**
		Conductivity	1	1.007	3.393	0.067
		Residuals	246	73.002		

df = degrees of freedom, *SS* = sum of squares, significant effects ($p<0.05$) are in bold. Tables generated with Type-II SS from the *car* package. Models represent best-fits from AIC model selection.

MOLECULAR ECOLOGY

Table S4. Summary of one-way analysis of variance models using spatial and environmental variables as predictors of Shannon diversity in water-borne bacterioplankton and four zooplankton taxa collected in six mountain lakes across five sampling time points. Models are ordered by the strength of relationships evaluated by Adjusted-R^2 values with select effects plotted in Figure 5.

Diversity metric	*Sample type*	*Effect*	*df*	*Residual-SE*	*Adjusted-R^2*	*Pr(>F)*
**Shannon Diversity**	*Water*	Latitude	1, 26	0.252	0.057	0.117
		Dissolved oxygen	1, 26	0.263	-0.027	0.600
		Conductivity	1, 26	0.264	-0.036	0.823
	Zooplankton	Latitude	1, 251	0.568	0.245	**<0.001**
		Dissolved oxygen	1, 251	0.595	1.172	**<0.001**
		Conductivity	1, 251	0.599	0.160	**<0.001**

df = degrees of freedom in numerator and denominator of one-way analysis; significant effects ($p<0.05$) are in bold. Main effects were chosen according to their inclusion in best-fit models) for free-living microbial communities, zooplankton-associated communities, or both (*Table* S3).

MOLECULAR ECOLOGY

Table S5. Analysis of variance tables testing environmental factors as predictors of relative abundance of bacteria classes in water-borne bacterioplankton and four zooplankton taxa collected in six mountain lakes across five sampling time points.

Bacteria taxonomic class	*Sample type*	*Effect*	*df*	*SS*	*F-value*	*Pr(>F)*
**Gammaproteobacteria**	*Water*	Latitude	1	0.026	17.213	**<0.001**
		Temperature	1	0.015	9.997	**0.005**
		Chlorophyll	1	0.010	6.464	0.019
		pH	1	0.006	4.064	0.056
		Conductivity	1	0.024	16.324	**<0.001**
		Residuals	22	0.033		
	Zooplankton	Latitude	1	2.949	74.393	**<0.001**
		Elevation	1	1.899	47.897	**<0.001**
		Temperature	1	0.096	2.427	0.121
		Chlorophyll	1	0.228	5.746	**0.017**
		pH	1	0.094	2.373	0.125
		Dissolved Oxygen	1	1.032	26.045	**<0.001**
		Conductivity	1	0.970	24.463	**<0.001**
		Residuals	240	9.513		
**Alphaproteobacteria**	*Water*	Latitude	1	0.007	4.778	**0.042**
		Elevation	1	0.024	15.051	**0.001**
		Temperature	1	0.004	2.453	0.135
		Dissolved oxygen	1	0.007	4.407	0.050
		Residuals	18	0.028		
	Zooplankton	Dissolved oxygen	1	0.231	5.911	**0.017**
		Conductivity	1	0.153	3.915	0.050
		Residuals	115	4.488		
**Actinobacteria**	*Water*	Conductivity	1	0.074	19.796	**<0.001**
		Residuals	24	0.089		
	Zooplankton	Residuals	6	0.001	NA	NA
**Bacteroidia**	*Water*	Latitude	1	0.060	9.790	**0.005**
		Elevation	1	0.028	4.597	**0.042**
		Dissolved oxygen	1	0.053	8.680	**0.007**
		Conductivity	1	0.036	5.821	**0.024**
		Residuals	23	0.141		
	Zooplankton	Latitude	1	0.965	25.149	**<0.001**
		Elevation	1	1.120	29.205	**<0.001**
		Temperature	1	0.179	4.674	**0.032**
		Dissolved oxygen	1	0.346	9.015	**0.003**
		Conductivity	1	0.991	25.825	**<0.001**
		Residuals	186	7.134		

df = degrees of freedom, *SS* = sum of squares, significant effects ($p<0.05$) are in bold. Tables generated with Type-II SS from the *car* package. Models represent best-fits from AIC model selection. *NA* represent no outputs from Intercept-only best-fit models (Actinobacteria-Zooplankton).

MOLECULAR ECOLOGY

Table S6. Summary of one-way analysis of variance models using spatial and environmental variables as predictors of bacteria taxa relative abundance in water-borne bacterioplankton and four zooplankton taxa collected in six mountain lakes across five sampling time points. Models are ordered by the strength of relationships evaluated by Adjusted-R^2 values with select effects plotted in Figure 5.

Bacteria taxonomic class	*Sample type*	*Effect*	*df*	*Residual-SE*	*Adjusted-R^2*	*Pr(>F)*
**Gamaproteobacteria**	*Water*	Conductivity	1, 26	0.054	0.436	**<0.001**
		Latitude	1, 26	0.064	0.222	**0.007**
	Zooplankton	Latitude	1, 246	0.220	0.073	**<0.001**
		Conductivity	1, 246	0.226	0.016	**0.025**
**Alphaproteobacteria**	*Water*	Elevation	1, 21	0.046	0.284	**0.005**
		Conductivity	1, 21	0.047	0.250	**0.009**
		Temperature	1, 21	0.050	0.145	**0.041**
	Zooplankton	Dissolved oxygen	1, 116	0.200	0.111	**<0.001**
		Conductivity	1, 116	0.202	0.096	**<0.001**
**Actinobacteria**	*Water*	Conductivity	1, 24	0.061	0.429	**<0.001**
		Elevation	1, 24	0.071	0.216	**0.010**
	Zooplankton	Conductivity	1, 5	0.015	-0.140	0.630
		Elevation	1, 5	0.014	-0.139	0.627
**Bacteroidia**	*Water*	Dissolved oxygen	1, 26	0.092	0.561	**<0.001**
		Latitude	1, 26	0.103	0.449	**<0.001**
		Conductivity	1, 26	0.126	0.183	**0.013**
	Zooplankton	Temperature	1, 190	0.209	0.019	**0.030**
		Dissolved oxygen	1, 190	0.211	-0.004	0.683
		Latitude	1, 190	0.211	-0.001	0.379

df = degrees of freedom in numerator and denominator of one-way analysis; significant effects (p<0.05) are in bold. Main effects were chosen according to their inclusion in best-fit models free-living microbial communities, zooplankton-associated communities, or both (*Table S5*).

MOLECULAR ECOLOGY

Table S7. PERMANOVA results testing the effects of spatial and environmental variables on free-living bacterioplankton and zooplankton-associated microbiome community composition using a Bray-Curtis dissimilarity matrix.

Sample Type	*Environmental variable*	*df*	*SS*	R^2	*F*	*Pr(>F)*
Water	Latitude	1	1.402	0.186	8.194	**0.001**
	Elevation	1	1.249	0.166	7.300	**0.001**
	Temperature	1	0.426	0.057	2.492	**0.010**
	pH	1	0.093	0.012	0.543	0.943
	Conductivity	1	0.576	0.076	3.365	**0.001**
	Dissolved organic carbon	1	0.168	0.022	0.981	0.453
	Chlorophyll *a*	1	0.217	0.029	1.266	0.223
	Dissolved oxygen	1	0.161	0.021	0.941	0.483
	Residual	19	3.251	0.431		
	Total	27	7.543	1.000		
Zooplankton	Latitude	1	6.160	0.060	17.893	**0.001**
	Elevation	1	4.087	0.040	11.873	**0.001**
	Temperature	1	1.472	0.014	4.277	**0.001**
	pH	1	1.319	0.013	3.831	**0.001**
	Conductivity	1	1.701	0.017	4.942	**0.001**
	Dissolved organic carbon	1	0.889	0.009	2.583	**0.005**
	Chlorophyll *a*	1	1.037	0.010	3.013	**0.003**
	Dissolved oxygen	1	1.987	0.019	5.771	**0.001**
	Residual	244	83.996	0.818		
	Total	252	102.649	1.000		

df = degrees of freedom, *SS* = sum of squares, significant effects ($p<0.05$) are in bold.

MOLECULAR ECOLOGY

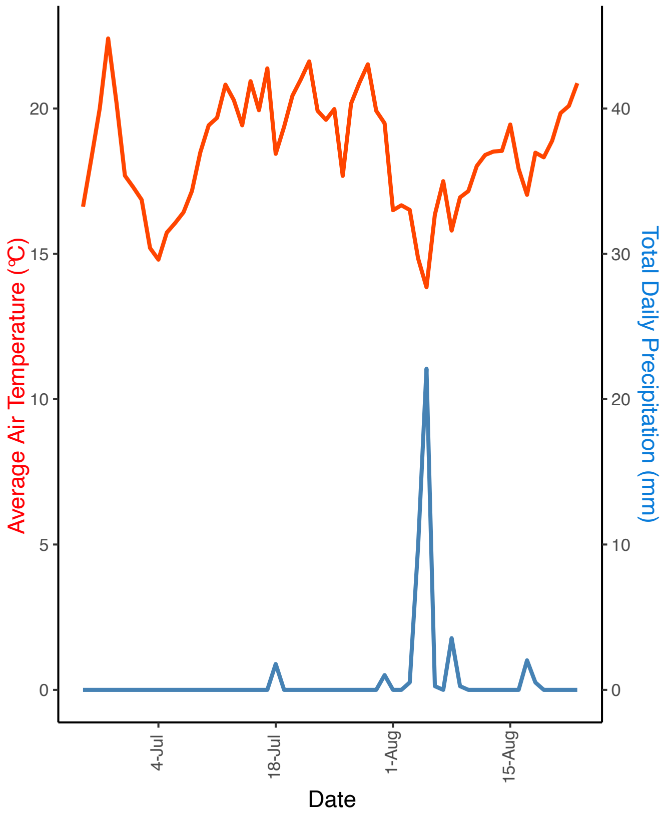

Figure S1. Average air temperature and total daily precipitation across the summer season in which lake sampling occurred. Data from the Sierra Nevada Research Station (SNARL) weather station in Mammoth Lakes, CA, USA.

MOLECULAR ECOLOGY

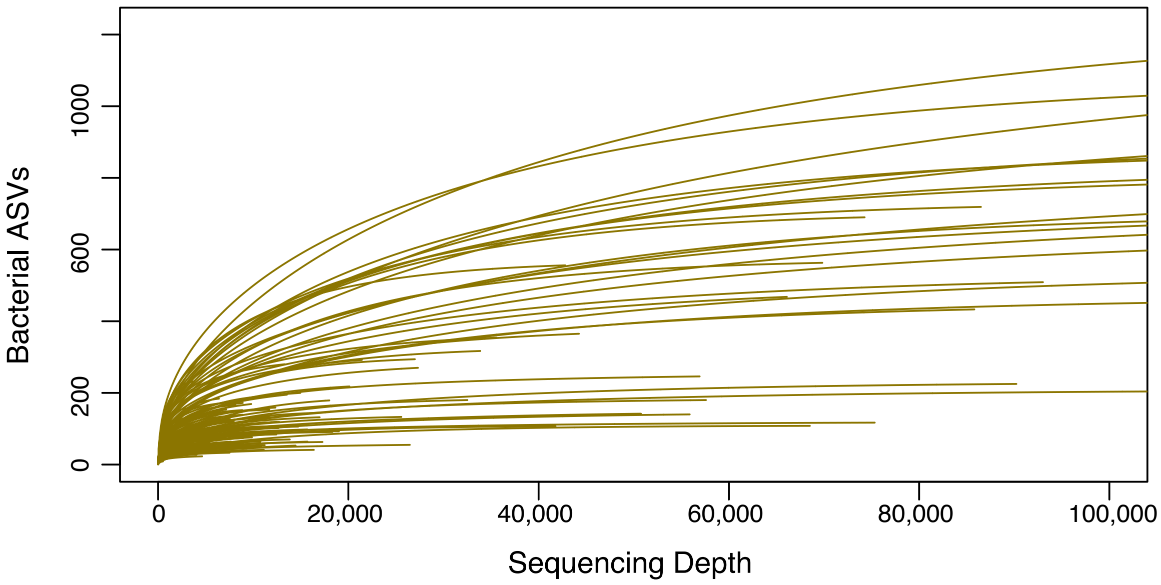

Figure S2. Rarefaction curve of number of bacterial ASVs as a function of sequencing depth (the number of reads per sample).

MOLECULAR ECOLOGY

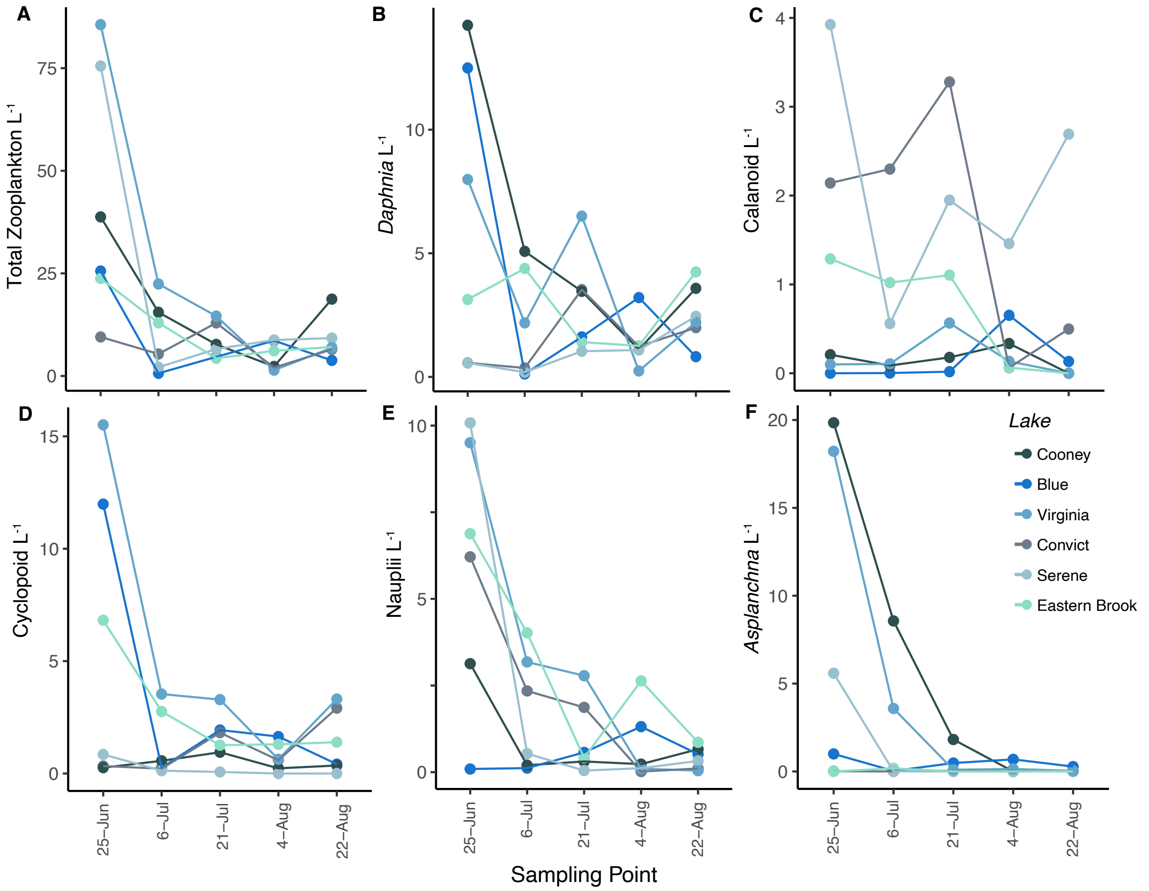

Figure S3. Changes in zooplankton densities across six mountain lakes across five sampling time points. (**A**) Total zooplankton densities across groups, and the densities of several abundance taxa, including (**B**) *Daphnia* cladocerans, (**C**) calanoid copepods, (**D**) cyclopoid copepods, (**E**) copepod nauplii, and (**F**) and *Asplanchna* rotifers.

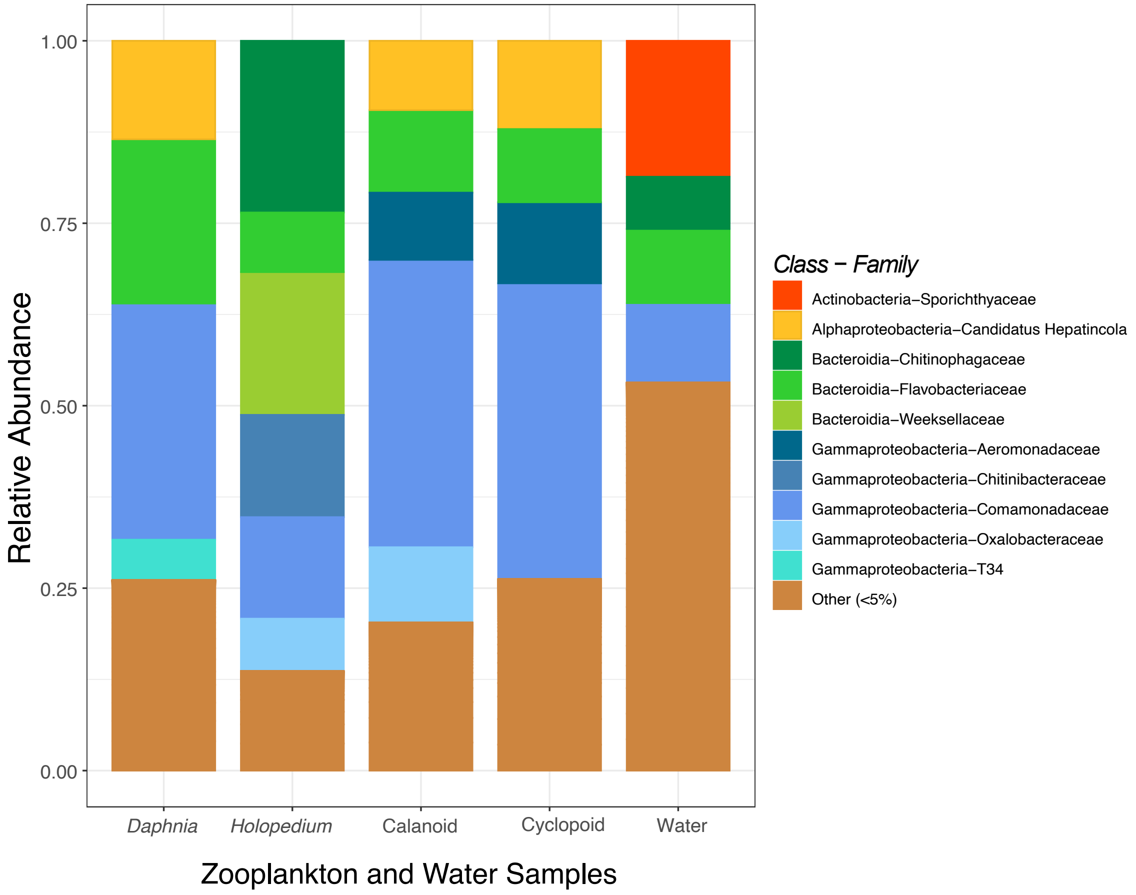

Figure S4. Relative abundance of bacterial taxa Families among *Daphnia*, *Holopedium*, calanoid and cyclopoid copepods, and bacterioplankton in water from lakes of the Eastern Sierra Nevada mountains. Samples were pooled across six lakes and five time points across the summer season.

MOLECULAR ECOLOGY

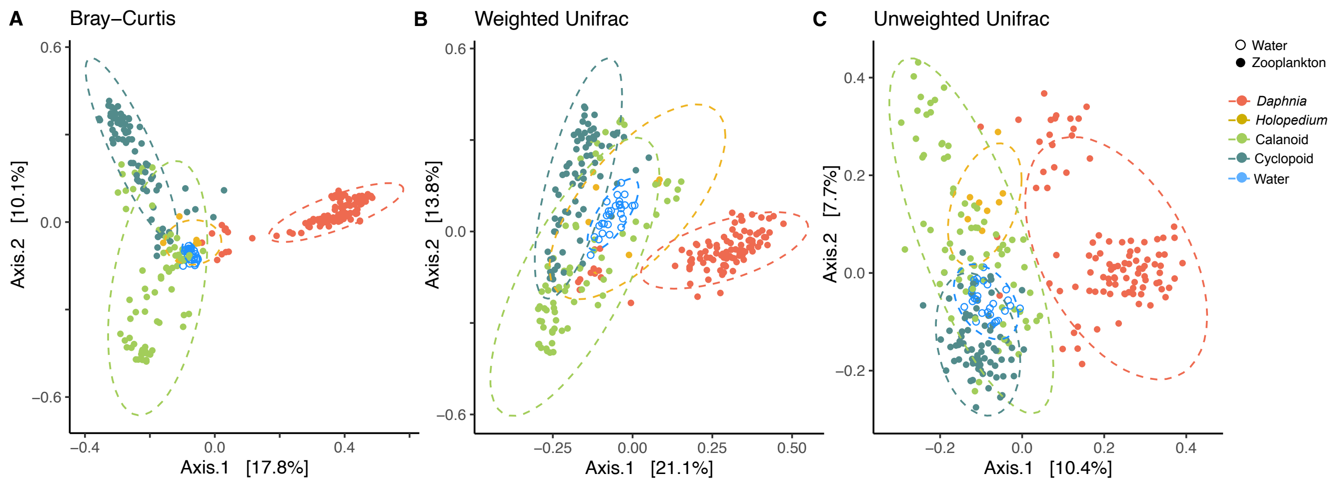

Figure S5. Analysis of zooplankton-associated and free-living (water) bacteria community beta diversity using (**A**) principal coordinate analysis (PCoA) of Bray-Curtis dissimilarity and (**B**) weighted and (**C**) unweighted unifrac distance. The percent of variation explained in PCoA-axes is in brackets. Ellipses represent 95% confidence intervals.
